# GPR81-mediated reprogramming of glucose metabolism contributes to the immune landscape in breast cancer

**DOI:** 10.1007/s12672-023-00709-z

**Published:** 2023-07-27

**Authors:** Xiaofeng li, Yiwen Chen, Ting Wang, Zifan Liu, Guotao Yin, Ziyang Wang, Chunxiao Sui, Lei Zhu, Wei Chen

**Affiliations:** 1 National Clinical Research Center for Cancer, Key Laboratory of Cancer Prevention and Therapy, Department of Molecular Imaging and Nuclear Medicine,Tianjin Medical University Cancer Institute and Hospital, Tianjin, China; 2 Department of Molecular Imaging and Nuclear Medicine, Tianjin Cancer Hospital Airport Hospital, Tianjin, China

**Keywords:** GPR81, Breast cancer, Glucometabolic reprogramming, Immune landscape

## Abstract

**Background:**

Local tumor microenvironment (TME) plays a crucial role in immunotherapy for breast cancer (BC). Whereas, the molecular mechanism responsible for the crosstalk between BC cells and surrounding immune cells remains unclear. The present study aimed to determine the interplay between GPR81-mediated glucometabolic reprogramming of BC and the immune landscape in TME.

**Materials and Methods:**

Immunohistochemistry (IHC) assay was first performed to evaluate the association between GPR81 and the immune landscape. Then, several stable BC cell lines with down-regulated GPR81 expression were established to directly identify the role of GPR81 in glucometabolic reprogramming, and western blotting assay was used to detect the underlying molecular mechanism. Finally, a transwell co-culture system confirmed the crosstalk between glucometabolic regulation mediated by GPR81 in BC and induced immune attenuation.

**Results:**

IHC analysis demonstrated that the representation of infiltrating CD8^+^ T cells and FOXP3^+^ T cells were dramatically higher in BC with a triple negative (TN) subtype in comparison with that with a non-TN subtype (P < 0.001). Additionally, the ratio of infiltrating CD8^+^ to FOXP3^+^ T cells was significantly negatively associated with GPR81 expression in BC with a TN subtype (P < 0.001). Furthermore, GPR81 was found to be substantially correlated with the glycolytic capability (P < 0.001) of BC cells depending on a Hippo-YAP signaling pathway (P < 0.001). In the transwell co-culture system, GPR81-mediated reprogramming of glucose metabolism in BC significantly contributed to a decreased proportion of CD8^+^ T (P < 0.001) and an increased percentage of FOXP3^+^ T (P < 0.001) in the co-cultured lymphocytes.

**Conclusion:**

Glucometabolic reprogramming through a GPR81-mediated Hippo-YAP signaling pathway was responsible for the distinct immune landscape in BC. GPR81 was a potential biomarker to stratify patients before immunotherapy to improve BC’s clinical prospect.

**Supplementary Information:**

The online version contains supplementary material available at 10.1007/s12672-023-00709-z.

## Introduction

Breast cancer (BC) is one of the most common malignancies in women worldwide, ranking first in morbidity and second in mortality, respectively [1]. Treatment strategies based on molecular subtype classification significantly improved the therapeutic efficacy of treatment for BC in recent decades [2]. However, the heterogeneity within each molecular subtype of BC and the development of treatment resistance warrant further investigation to achieve individualized therapy and precision medicine [3, 4]. As a promising treatment, immunotherapy, particularly for immune checkpoint blockade (ICB) therapy, is increasingly accepted and applied in clinical practice for cancer [5]. Clinical trials involving ICB for BC focus on a subpopulation of BC with a triple negative (TN) subtype or a human epidermal growth factor receptor-2 (HER2) positive subtype [6, 7], and the local tumor microenvironment (TME) is considered a pivotal determinant of therapeutic efficiency and prognosis [8]. Tumor-infiltrating lymphocytes (TILs), as dominant cell components of host immune response against tumors in TME, play a predictive and prognostic role in BC with ICB therapy [9]. A higher TIL score and a higher level of programmed cell death-ligand 1 (PD-L1) expression were found to be significantly correlated with a higher pathological complete response (pCR) rate and better outcome of neoadjuvant immunotherapy for BC with a TN or a HER2 ( +) subtype [10, 11]. In addition, regarding the dominant BC subpopulation with a Luminal subtype, a higher TIL score was also associated with an increased pCR and improved survival, particularly for a subgroup with Ki-67 ≥ 20% [12]. Although TIL score and PD-L1 expression are suggested as promising indexes to select potentially responsive BC patients to ICB therapy, their translation to clinical practice is limited by non-standardization, complexity and subjectivity in TIL and PD-L1 evaluation. A more feasible biomarker is urgently needed to address this issue.

Determination of the underlying mechanism for induced immunosuppression in TME holds promise to remarkably improve the efficiency of tumor immunotherapy [13]. As two important hallmarks of tumor, immunosuppression and glycolytic reprogramming are closely related [14, 15]. From a metabolism perspective, tumorigenesis and tumor development are considered disease processes of metabolic reprogramming [16]. As reported, various types of tumors were characterized by enhanced glycolysis which led to an accumulation of lactate in TME, including BC [17]. Lactate, previously regarded as a metabolic waste from glycolysis, is increasingly recognized as a fuel for tumor metabolism and a regulatory molecule involved in tumor intracellular signaling [18]. Furthermore, lactate is also found to be able to attenuate anti-tumor immunity and induce tumor immunosuppression [19]. However, the role of lactate in the metabolic regulation of BC and the underlying molecular mechanism remain unclear. Moreover, whether the modulation of metabolic status of BC contributes to the remodeling of immune landscape in local TME, which is worthy of being investigated as the main purpose in this study.

G-protein-coupled receptors 81 (GPR81), as an endogenous receptor for lactate, is recently becoming a research interest in tumor metabolism and tumor immunity [20, 21]. As previously reported, GPR81 was crucial for tumor development and progression in various types of tumors, such as lung cancer, pancreatic cancer and breast cancer [22–24]. Moreover, GPR81-mediated tumor immunosuppression is suggested as a novel mechanism involved in the complicated immunoregulatory network in tumors [22, 25]. Considering breast cancer, the role of GPR81 in the regulation of glucose metabolism [21, 24] and its association with tumor immunoregulation [25], especially for tumor immune escape, are worthy of being deeply explored. In the present investigation, human BC microarray immunohistochemical (IHC) staining was first performed to detect the association between GPR81 expression and TIL status. Then, several established stable BC cell lines with downregulation of GPR81 were used in this investigation to directly identify the role of GPR81 in glycolysis reprogramming and underlying molecular mechanisms. Last but not least, a transwell co-culture system was applied to ascertain the interaction between the regulation of glucose metabolism mediated by GPR81 in BC and induced immune attenuation and immune escape in TME. Promisingly, GPR81 will be helpful as a biomarker to effectively screen potentially responsive BC subpopulation for ICB therapy or other immunotherapy. Metabolism intervention against GPR81 is expected to be applied in a combination treatment with ICB therapy to further improve the prospect of tumor immunotherapy for BC [26, 27].

## Materials and methods

### Cell culture and blood sample collection

A total of three human BC cancer cell lines were used in this study, including T47D, SK-BR-3 and MDA-MB-231, which represent BC with a Luminal subtype, a HER2 ( +) subtype, and a TN subtype, respectively. All of these cell lines were provided by the Key Laboratory of Cancer Immunology and Biotherapy in our institution. The cells were regularly cultured in Dulbecco's modified eagle medium (DMEM) supplemented with 10% fetal bovine serum (FBS) at 37 °C in a 5% CO_2_ incubator. Human peripheral blood samples were obtained from healthy volunteer blood donors (Blood Center in our city) to isolate peripheral blood mononuclear cells (PBMCs) by centrifugation on Ficoll density gradients (GE Healthcare Life Sciences, Shanghai, China). Freshly obtained PBMCs were then incubated and activated in 10% FBS Roswell Park Memorial Institute (RPMI) 1640 mediums and used in the following transwell co-culture.

### Tissue microarrays and immunohistochemistry analysis

Tissue microarrays (TMA) of BC used in the present study were provided from a domestic company (SHANGHAI OUTDO BIOTECH CO., LTD. Shanghai, China), and this company also supported the streptavidin–biotin-peroxidase staining. IHC assay was performed on those TMA slides to determine the status of molecular subtype, the level of GPR81 expression and the local immune landscape. A total of 199 pathologically confirmed BC specimens in paraffin blocks, including 101 Luminal, 36 HER2 ( +) and 62 TN, were collected and used for making tissue microarray chips. To enable the characterization of the local immune landscape in the BC tissue microarray chips, we selected 2 mm as the pore diameter in the micro-array chips. Briefly, TMA slides were first deparaffinized and rehydrated according to typical protocols. Then, the slides were pretreated with microwaves, blocked, and incubated with a series of primary antibodies overnight at 4 °C according to the manufacturer’s instructions, followed by incubation with appropriate horseradish peroxidase (HRP)-conjugated secondary antibodies (1:2000; Santa Cruz Biotechnology, Inc., Dallas, TX) for 1 h at room temperature. Finally, signals on the slides were revealed using 3,3-diaminobenzidine (DAB) buffer as substrate. In place of primary antibodies for the negative control, PBS was used. With respect to IHC analysis for molecular subtype of BC, specific primary antibodies against estrogen receptor (ER), progesterone receptor (PR), HER2 (1:200, Cell Signaling Technology, Beverly, MA, USA) and GPR81 (1:200, Abcam, Cambridge, UK) were applied. The molecular subtypes of BC were categorized as follows: Luminal (ER positive and/or PR positive), HER2 enriched (ER negative, PR negative and HER2 positive), and TN (ER negative, PR negative, HER2 negative). For TIL immunohistochemistry and quantification, primary specific monoclonal antibodies against CD8 (Biolegend, San Diego, CA) and forkhead box P3 (FOXP3) (1:100, Abcam, Cambridge, UK. ab20034, clone 236A/E7) were used. TIL densities in the TMA slides of BC were evaluated microscopically at high power (× 40 objective lens), and ten high-power fields of each case, including five tumor beds and five peripheries, were selected for digital photographs. Then the total numbers of CD8^+^ and FOXP3^+^ TILs in each field and the ratio of CD8 to FOXP3 were counted manually and confirmed by two specialized pathologists blinded to the clinicopathologic characteristics of BC patients.

### Immune scores calculation for breast cancer patients based on molecular subtype classification

Ribonucleic acid (RNA)-sequencing data of BC patients from the public database (The Cancer Genome Atlas, TCGA) were first standardized for further analysis. Then, immune score calculation for BC was performed using gene set enrichment analysis (GSEA). Briefly, based on the molecular subtype classification of BC, the included BC patients from the TCGA database were first divided into three categories (Luminal, HER2 and TN). Then, the gene set variation analysis (GSVA) package was used for a single sample GSEA (ssGSEA) of 29 immune-associated gene sets chosen to represent tumor immunity. In the end, a heat map was drawn to visualize the distinct immune characteristics of different molecular subtype groups based on the ssGSEA scores for various tumor-infiltrating immune cells. Furthermore, violin plots were also drawn to highlight the differences in of TIL, CD8^+^ T cell infiltration and regulatory T cell (Treg) infiltration scores between different subgroups.

### Establishment of stable breast cancer cell lines with down-regulated expression of GPR81 by lentiviral transduction

Conventional lentiviral transduction technology was used to modulate GPR81 expression in BC cell lines. Before lentiviral packaging and transduction, a small hairpin ribonucleic acid (shRNA) targeting GPR81 mRNA sequence and a control shRNA were cloned into pLL3.7 (Suzhou Genepharma Co., Ltd, Nanjing, China) vectors, respectively. The shRNA sequences targeting GPR81 were synthesized at Suzhou Genepharma Gene Technology (Suzhou Genepharma Co., Ltd, Nanjing, China). Lentiviral particles were produced by transient co-transfection of HEK293T cells with pSPAX2, pMD2.G vectors plus pLL3.7-GPR81-shRNA or control shRNA lentiviral vectors. The lentiviral particles produced were collected from the culture supernatants 48 h afterward, and the viral titer was determined by transducing HEK293T cells with successive dilutions of the supernatants. For the lentiviral transduction of BC cells, one single round of transduction with lentiviral particles was performed at a multiplicity of infection (MOI) of 5 in the presence of 5 μg/ml PolyBrene (Sigma-Aldrich, Oakville, Canada). Subsequently, BC cells were continually cultured in a complete medium with an addition of 2 μg/ml of puromycin for 5–8 days to establish stable BC cell lines with downregulation of GPR81. The levels of GPR81 expression were verified using western blotting assays before further phenotypic and functional assays.

### Evaluation of the glycolytic capacity of BC cells in vitro

Measurement of glycolytic capacities of BC cell lines with different subtypes and established stable BC cells with downregulation of GPR81 were achieved through incubating BC cell lines with glucose analog ^18^F-fluorodeoxyglucose (FDG) in vitro and determining the concentrations of lactate secreted by BC cells lines in cell culture supernatants. Briefly, BC cells were seeded into 12-well plates in triplicate and cultured for 6 h, 1 ml fresh medium containing 10 μCi ^18^F-FDG (synthesized in our laboratory) were added to each well for different durations (0.5, 1 and 2 h). Following rinsing with (phosphate buffer saline) PBS, single-cell suspensions were prepared in PBS to detect the uptake of ^18^F-FDG by BC cells through the radioactivity counting with a γ-radiation counter (Cobra Quantum; Packard). The results were normalized to the radioactivity of 1 × 10^5^ cells. According to the manufacturer’s instructions, lactate produced by BC cells was collected and measured using a lactate assay kit (Solarbio® Life Science, Beijing, China). The results for lactate production were expressed as ratios of the values in the test group to the values in the control group.

### Western blotting assay

To reveal the molecular mechanism responsible for GPR81 mediated regulation of glycolysis in BC cell lines, we performed a western blotting assay to detect the correlation between GPR81 expression and the activation status of Hippo- Yes-associated protein (YAP) signaling. Briefly, BC cell lines with different subtypes and established stable BC cells with down-regulated GPR81 were lysed using radioimmunoprecipitation assay (RIPA) buffer with an addition of protease/phosphatase inhibitor cocktail (Cell Signaling Technology, Beverly, MA, USA). Subsequently, equal amounts of proteins (30–50 μg/lane) from each sample were loaded and separated by 10% sodium dodecylsulfate-polyacrylamide gel electrophoresis (SDS-PAGE), and then the proteins separated in gels were transferred onto polyvinylidene difluoride (PVDF) membranes. The membranes were incubated with specific primary antibodies overnight at 4 °C to detect specific proteins or their activation status after being blocked with 5% skimmed milk powder at room temperature for 1 h. Following incubation, membranes were rinsed with tris-buffered saline (TBS) containing 0.1% Tween 20 and finally incubated with horseradish peroxidase (HRP) conjugated secondary antibodies (1:2000; Santa Cruz Biotechnology, Inc., Dallas, TX) at room temperature for 1 h. The signals on the membrane were developed and visualized on a western blotting detection system (Syngene G:BOX XT4, Gene Company Limited, HongKong, China) using an enhanced chemiluminescence (ECL) reagent (Millipore, Bedford, MA). The primary antibodies used in this investigation were all from Cell Signaling Technology (1:1000, CST, Beverly, MA, USA), including primary antibodies against anti-p-the large tumor suppressor (LATS1)/LATS1, anti-p-YAP/YAP, except for GPR81 (1:1000, Abcam, Cambridge, UK).

### Lactate stimulation assay and a transwell co-culture system in vitro

A transwell co-culture system was used to imitate the interplay between BC cells and surrounding immune cells in vivo. Briefly, freshly obtained PBMCs were seeded into the upper chambers of a transwell co-culture system and activated in 10% FCS DMEM under stimulation with anti-CD3, anti-CD28 (1 μg/ml, Biolegend, San Diego, CA) plus interleukin 2 (IL-2, 10 ng/ml, Biolegend, San Diego, CA) in the presence of transforming growth factor β1 (TGF-β1, 10 ng/ml, Biolegend, San Diego, CA). Whereas, established stable BC cells differing in GPR81 expression were seeded into the lower chambers. The upper chamber was inserted into the lower chamber, and the filter membrane at the bottom of the upper chamber was immersed in the medium of the lower chamber. In this case, though direct contact between BC cells and immune cells was blocked by the separation design of upper chamber and lower chamber, the communication of molecules in the medium between BC cells in the lower chamber and immune cells in the upper chamber was available via the filter membrane. For the lactate stimulation assay, sodium lactate (20 mM, Sigma-Aldrich, Saint Louis, MO) was added to the above stimulating culture medium. Moreover, 2-Deoxy-d-glucose (2-DG, 5 mmol/L, ApexBio Tech LCC), a glycolysis inhibitor, was added into the transwell co-culture system to determine the role of enhanced glycolysis mediated by GPR81 in immunoregulation. Following three days of co-culture, PBMCs in the upper chamber were collected to identify the changes in percentages of different lymphocyte subsets using flow cytometry.

### Flow cytometric analyses

Flow cytometric assay was performed to identify the changes in the frequencies of different lymphocyte subsets from PBMCs cultured in a transwell co-culture system. Briefly, PBMCs were directly incubated with fluorescence-labeled monoclonal antibodies specific to CD3, CD8 (Biolegend, San Diego, CA, USA) and appropriate isotype-matched control antibodies for 30 min on ice. Whereas, for forkhead box protein 3 (FOXP3) staining, which is a specific marker for regulatory T (Treg) cell, cells were first fixed and permeabilized using CytoFix/Cytoperm (eBioscience, San Diego, CA, USA) after an appropriate surface staining according to the manufacturer’s instructions, followed by staining for intracellular FOXP3 (eBioscience, San Diego, CA, USA). Data were acquired and analyzed on Fluorescence Activated Cell Sorting (FACS) Aria I (BD Biosciences, San Diego, CA, USA) by using CellQuest software (BD Biosciences, San Diego, CA, USA).

### ^18^F-FDG micro-PET/CT imaging of human BC xenograft models with different levels of GPR81 in nude mice

Human BC xenograft models were established by subcutaneously injecting the nude mice at the dorsum of the left axilla with human BC cells, including the control MDA-MB-231 cells and the developed MDA-MB-231 cells with downregulation of GPR81 used in the transwell co-culture system mentioned above. To guarantee the success of the xenograft establishment, 2 × 10^6^ MDA-MB-231 cells at the logarithm growth period were collected and suspended in 100 ul sterile PBS for each animal model. Xenograft growth was monitored three times weekly until a maximal diameter of the xenograft reached 8–10 mm. ^18^F-FDG micro-Positron Emission Tomography/Computed Tomography (PET/CT) imaging (IRIS PET/CT, Inviscan, France) was performed after intravenous injection of ^18^F-FDG at a concentration of 5 μCi/g to determine the accumulation of the glucose analog ^18^F-FDG in xenograft in vivo. Unlike to clinical human ^18^F-FDG PET/CT imaging, micro-PET imaging was performed first and followed by micro-CT imaging. The whole scan process almost took 10 min for each animal. The PET/CT images were then constructed with attenuation correction. The maximal standard uptake value (SUVmax) of the xenograft was obtained as the main outcome measurement.

### Statistical analyses

Data were presented as the mean ± standard deviation (SD) for percentages in this study. Student’s unpaired t-test and one-way analysis of variance (ANOVA) were performed to determine the significance of differences between group means using the GraphPad Prism software version 5.0 (GraphPad, La Jolla, CA). Correlations between GPR81 expression and molecular subtypes or other clinicopathological factors were tested by Spearman’s rank correlation analysis. A P-value under 0.05 was considered to indicate a statistically significant difference.

## Results

### Correlation between GPR81 and molecular subtype classification of BC

**Fig. 1:**
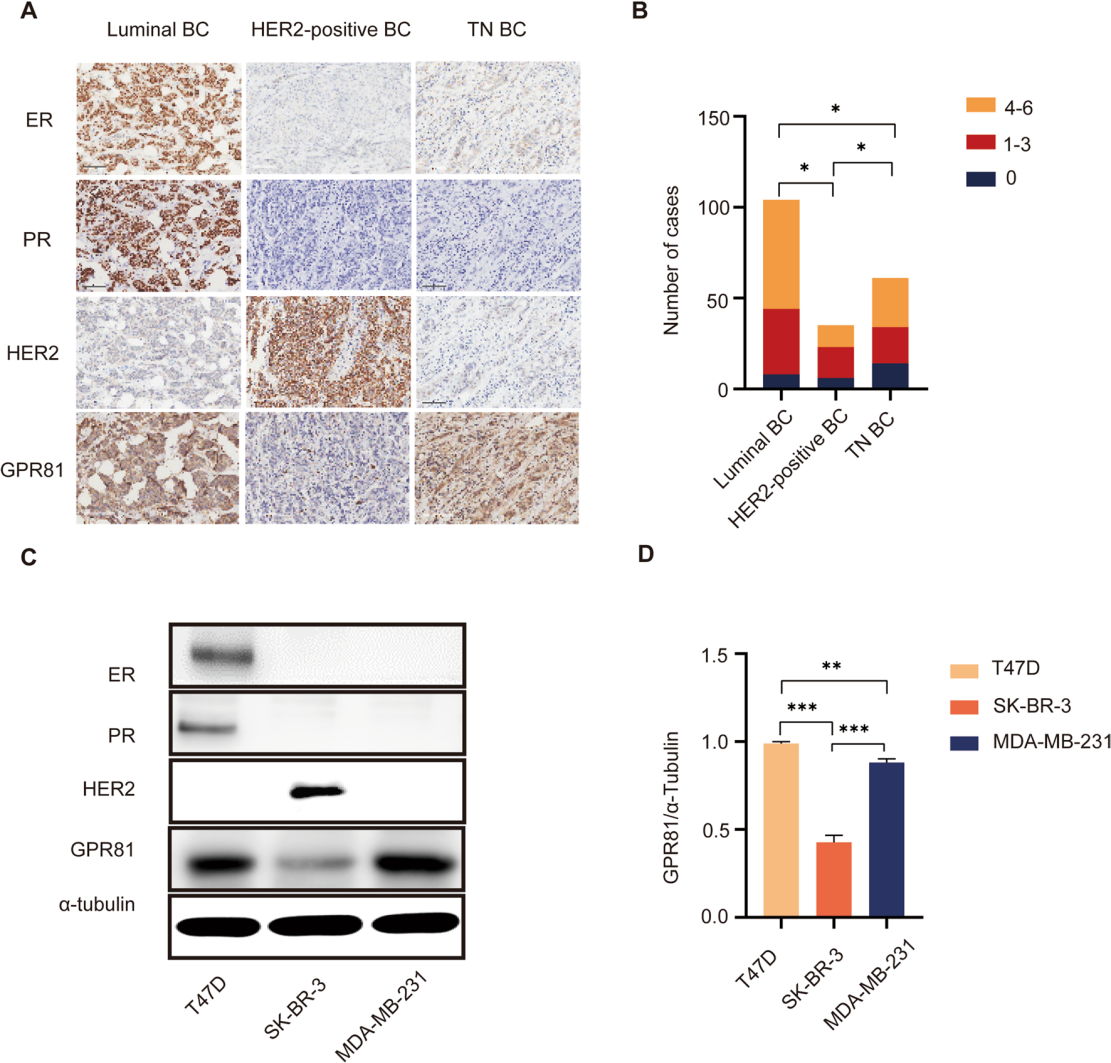
Distinct GPR81 expressions among BC with different molecular subtypes. **A** IHC assay was used to evaluate the molecular subtype classification and the level of GPR81 expression in BC. Representative IHC profiles were shown for BC with different molecular subtypes, including a Luminal subtype (ER + , PR + , HER2-), a HER2 + (ER-, PR-, HER2 +) and a TN subtype (ER -, PR-, HER2-). **B** Histogram was drawn to summarize the levels of GPR81 expression in individual BC patients based on molecular subtype classification. As shown, BC with a Luminal subtype tended to have a higher level of GPR81 in contrast with BC with a non-Luminal subtype. **C** The expression levels of GPR81 were assessed by western blotting assay in different BC cell lines with different molecular subtypes. **D** Histogram was depicted to summarize the ratio of GPR81 gray values to ɑ-tubulin gray values based on the results from three independent experiments. Consistently, BC cell lines with a Luminal subtype (T47D) or a TN subtype (MDA-MB-231) was shown with a significant enrichment of GPR81. *p < 0.05, **p < 0.01, ***p < 0.001

Firstly, BC microarray immunohistochemical (IHC) staining was performed to determine the correlation between GPR81 and the molecular subtype classification of BC. As shown in Fig. 1A and Fig. 1B, the percentages and the intensities of GPR81 positively stained cells were higher and stronger in BC with a Luminal subtype than in BC with a HER2 or a TN subtype. Regarding the difference of GPR81 expression between HER2 subgroup and TN subgroup, the latter was found to be with a relatively higher GPR81 level. Moreover, BC cell lines with different molecular subtypes were also used to detect the association between GPR81 expression and molecular subtype classification of BC. Consistently, results from western blotting assay demonstrated distinct GPR81 levels in BC cell lines with different molecular subtypes, with significant enrichment of GPR81 in T47D (Luminal) cell line and MDA-MB-231 cell line (TN) (Fig. 1C, D).

### Association between GPR81 level on tumor cells and tumor-infiltrating immune cells status in BC with different molecular subtypes

**Fig. 2:**
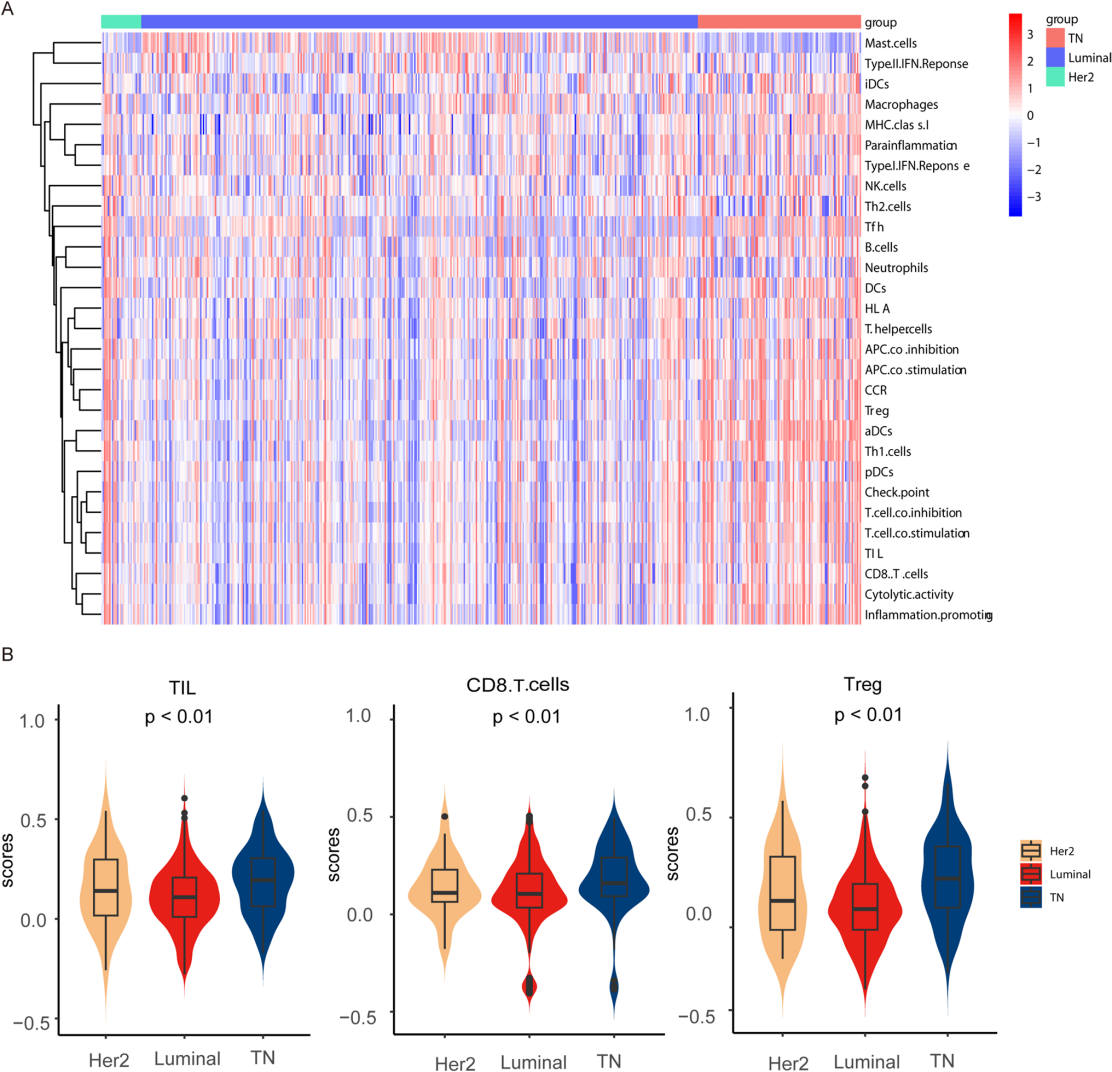

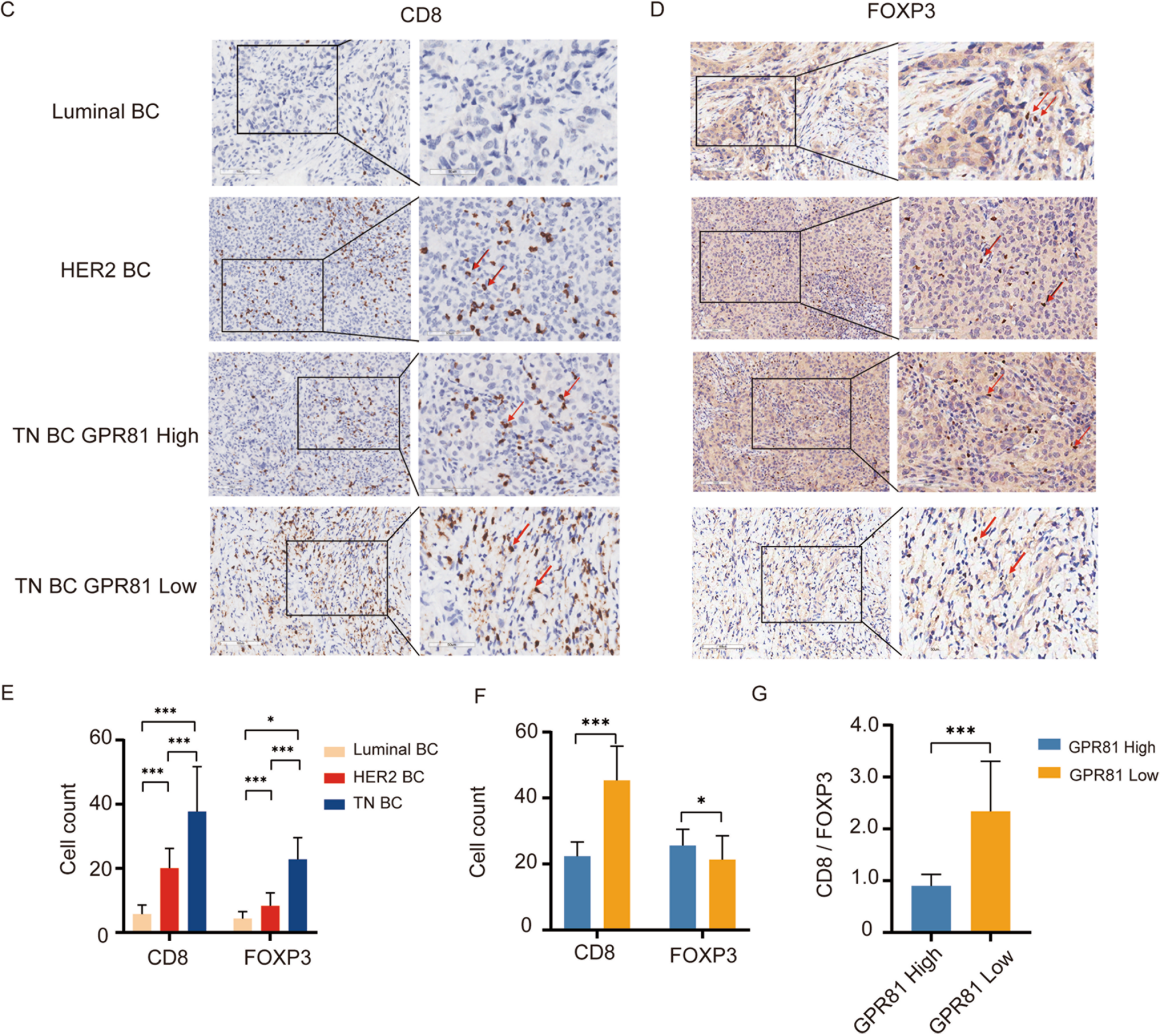
The tumor-infiltrating immune cell status in BC was significantly correlated with the level of GPR81 expression. **A** A bioinformatic analysis based on the TCGA-BC transcriptome data was first performed to detect the association between molecular subtype and immune scores in BC. As illustrated in the heat map, BC with a HER2 or a TN subtype was characterized by higher immune scores of a variety of tumor-infiltrating immune cells compared to that with a Luminal subtype, suggesting relatively higher immunogenicity of BC with a non-Luminal subtype in contrast with that with a Luminal subtype. **B** Based on the bioinformatic analysis, the violin blots indicated that the TIL, CD8^+^ T and Treg cells infiltration scores in the HER2 or TN subtype group were markedly higher than in the Luminal subtype group. The potential role of GPR81 on BC cells in immune responses against BC was revealed by IHC assay. A higher representation of lymphocyte infiltration in BC tissues, including CD8^+^ T cells **C** and FOXP3^+^ T cells **D**, was revealed by IHC assay. **E** As summarized in the histogram, a remarkably higher infiltration for both CD8^+^ T cells and FOXP3^+^ T cells was found in BC with a TN subtype in comparison with that with a non-TN subtype. **F** Particularly for BC with a TN subtype, the infiltration of CD8^+^ T cells was negatively correlated with the level of GPR81 expression, whereas the status of FOXP3^+^ T cell infiltration was positively associated with the level of GPR81 expression. Both representative IHC profiles **C**, **D** and a summarization of the numbers of infiltrating lymphocytes in individuals were shown. **G** For the ratio of infiltrating CD8^+^ to FOXP3.^+^ Treg cells in BC with a TN subtype, a lower ratio was revealed for the GPR81-high subgroup in comparison with that of the GPR81-low subgroup, suggesting a potential immune attenuation induced by GPR81. *p < 0.05, **p < 0.01, ***p < 0.001

Given the correlation between GPR81 and the molecular subtype classification of BC, a bioinformatic analysis based on The Cancer Genome Atlas (TCGA) transcriptome data was first performed to assess the relationship between the molecular subtype classification of BC and the immune landscape in TME. As illustrated in the heat map (Fig. 2A), BC with a HER2 or TN subtype was characterized by a higher immune score compared to that with a Luminal subtype. Specifically, the general TIL score, CD8^+^ T cell infiltration score and regulatory T cells (Treg) score were found to be higher in BC with a TN subtype in comparison with that with a non-TN subtype (Fig. 2B). BC microarray IHC staining was performed in the study to verify the previous bioinformatic analysis results. As shown, the tumor-infiltrating CD8^+^ T cells and FOXP3^+^ T cells were significantly higher in BC with a TN subtype than that with a non-TN subtype (Fig. 2C, D, E). In addition, the representation of tumor-infiltrating T cells was markedly correlated with the GPR81 level, particularly for BC with a TN subtype. As illustrated in Fig. 2F for BC with a TN subtype, the percentages of infiltrating CD8^+^T cells decreased, whereas the percentages of FOXP3^+^ T cells increased in groups with a higher level of GPR81 compared to that with a lower level of GPR81. Consistently, the ratio of CD8 to FOXP3 was significantly negatively correlated with GPR81 expression in BC with a TN subtype (Fig. 2G).

### Reprogramming of glucose metabolism in BC mediated by GPR81

**Fig. 3:**
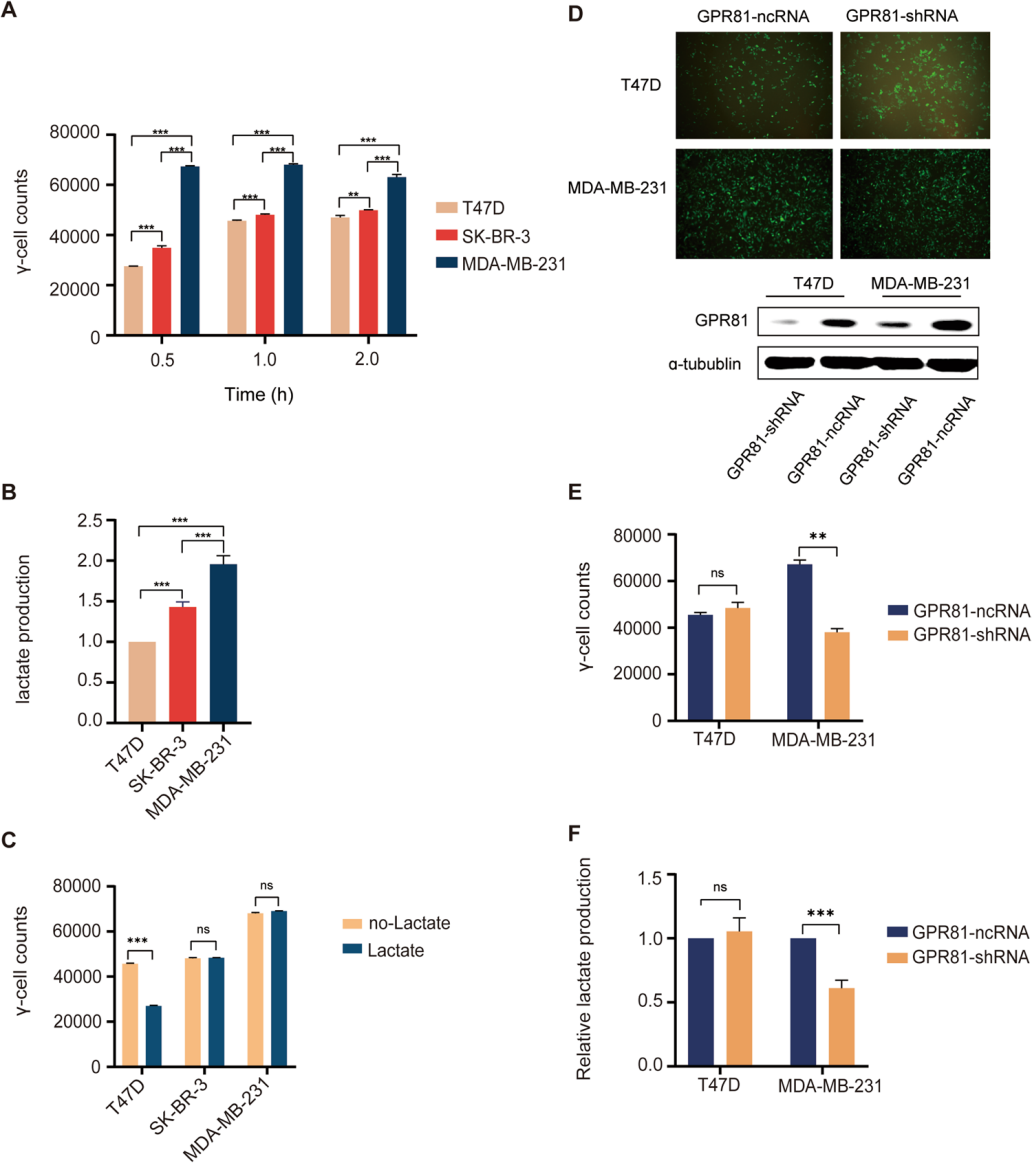
GPR81-mediated glycolytic regulation in BC cells with different molecular subtypes. **A** The uptake of glucose analog (^18^F-FDG) and lactate production by BC cells were measured to evaluate the glycolytic capacities of BC cells with different molecular subtypes. BC cells with a TN subtype (MDA-MB-231) tended to take in more ^18^F-FDG than that with a non-TN subtype (T47D, SK-BR-3). **B** Consistently,increased production of lactate was found in BC cells with a TN subtype compared to that with a non-TN subtype, suggesting enhanced glycolysis in BC cells with a TN subtype compared to that with a non-TN subtype. **C** Whereas, the lactate addition significantly attenuated the uptake of ^18^F-FDG by BC cells with a Luminal subtype (T47D), but slightly influenced that with a non-Luminal subtype (SK-BR-3, MDA-MB-231). **D** The establishment of stable T47D and MDA-MB-231 cells with down-regulated expressions of GPR81 through lentiviral transduction of GPR81-shRNA was validated by fluorescence microscopy and western blotting assays. **E**, **F** The role of GPR81 in reprogramming of glycolysis was directly assessed in established stable T47D and MDA-MB-231 cells with a down-regulated expressions of GPR81. For BC cells with a TN subtype (MDA-MB-231), downregulation of GPR81 expression (GPR81-shRNA) resulted in remarkably decreased glycolysis characterized by reduced uptake of.^18^F-FDG **E** and lactate production **F**. Whereas, for BC cells with a Luminal subtype (T47D), the glycolytic capacities were not significantly influenced by the downregulation of GPR81, suggesting distinct regulations of glucose metabolism mediated by GPR81 depended on intrinsic metabolic status of BC. *p < 0.05, **p < 0.01, ***p < 0.001

The glycolytic capacities of BC cells with different molecular subtypes were evaluated in vitro through measurement of uptake of glucose analog (^18^F-FDG) and lactate production by BC cells. As shown in Fig. 3A, B, more ^18^F-FDG uptake (Fig. 3A) and lactate production (Fig. 3B) are observed in BC cell line with a TN subtype (MDA-MB-231) in comparison with BC cell lines with a non-TN subtype (T47D, SK-BR-3), suggesting enhanced glycolysis in BC cells with a TN subtype in contrast with BC cells with a non-TN subtype. Whereas, the lactate addition significantly attenuated the capability of glucose uptake by BC cells with a Luminal subtype (T47D) but slightly influenced BC cells with a non-Luminal subtype (SK-BR-3, MDA-MB-231) (Fig. 3C). Stable T47D and MDA-MB-231 cells with down-regulated expressions of GPR81 were established through lentiviral transduction of GPR81-shRNA to directly assess the role of GPR81 in reprogramming of glycolysis (Fig. 3D). As demonstrated, MDA-MB-231 with a down-regulated GPR81 expression (GPR81-shRNA) exhibited remarkably decreased glycolysis characterized by reduced ^18^F-FDG uptake (Fig. 3E) and lactate production (Fig. 3F). In contrast, the capability of glycolysis in T47D cells was not significantly influenced by the downregulation of GPR81 (Fig. 3E, F), suggesting a distinct modulation of glucose metabolism mediated by GPR81 depending on intrinsic metabolic status of BC.

### Molecular mechanism responsible for the regulation of glucose metabolism mediated by GPR81 in BC

**Fig. 4:**
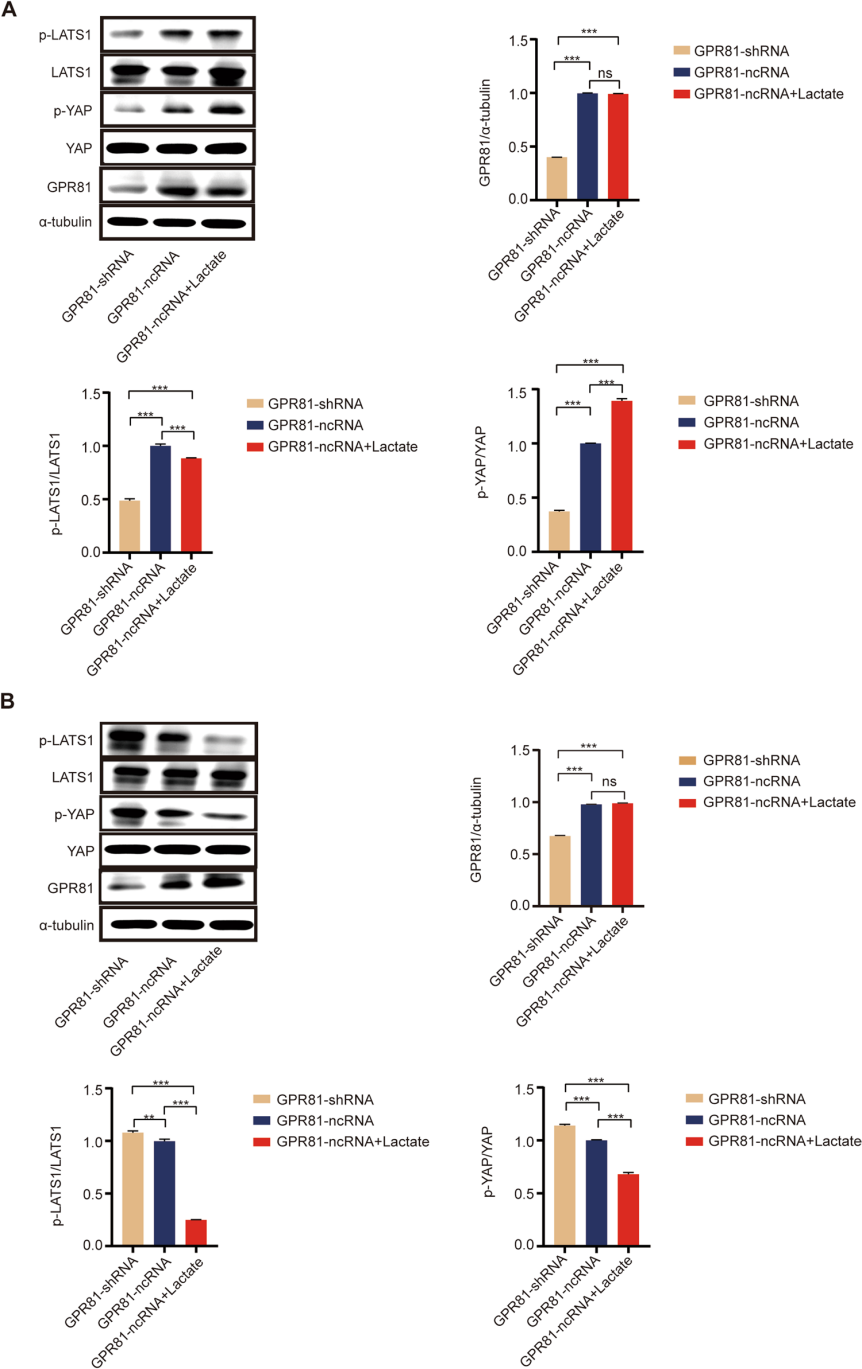
The Hippo-YAP signaling pathway was responsible for regulating glucose metabolism mediated by GPR81 in BC. The underlying molecular mechanisms for glycolytic reprogramming mediated by GPR81 in BC were detected by western blotting assay. **A** With sufficient glucose in the culture medium, inhibited activation of Hippo signaling with a decreased level of p-LATS1 and p-YAP was induced by downregulation of GPR81, and control T47D cells (GPR81-ncRNA) with lactate treatment was found to be with enhanced activation of Hippo signaling reflected by increased levels of p-LATS1 and p-YAP. **B** Whereas, for MDA-MB-231 cells, the phosphorylation levels of LATS1 (p-LATS1) and YAP (p-YAP) was remarkably increased in MDA-MB-231 cells with downregulation of GPR81 expression (GPR81-shRNA) compared to that in control MDA-MB-231 cells (GPR81-ncRNA), indicating enhanced activation of Hippo signaling induced by downregulation of GPR81. In addition, lactate treatment for control MDA-MB-231 cells (GPR81-ncRNA) resulted in attenuated activation of Hippo signaling which was reflected by decreased phosphorylation levels of LATS1 and YAP. A representative immunoblotting profile was shown on the upper, and the ratios of phosphorylated proteins to total proteins which were used to evaluate the degree of activation, were summarized based on results from three independent experiments (lower). *p < 0.05, **p < 0.01, ***p < 0.001

The underlying molecular mechanism for glycolytic reprogramming mediated by GPR81 in BC was detected by western blotting assay. In the presence of sufficient glucose, downregulated GPR81 led to inhibited Hippo signaling with decreased levels of p-LATS1 and p-YAP in T47D cells (Fig. 4A). Additionally, control T47D cells (GPR81-ncRNA) with lactate treatment was found to be with an enhanced Hippo-YAP signaling reflected by increased levels of p-LATS1 and p-YAP (Fig. 4A). Whereas, for MDA-MB-231 cells, increased phosphorylation levels of LATS1 (p-LATS1) and YAP (p-YAP) induced by downregulation of GPR81 indicated an enhanced activation of Hippo signaling by downregulated GPR81 (Fig. 4B). In contrast, lactate treatment for control MDA-MB-231 cells (GPR81-ncRNA) resulted in an attenuated activation of Hippo-YAP signaling which was reflected by decreased levels of p-LATS1 and p-YAP (Fig. 4B). To sum up, a Hippo-YAP dependent pathway based on the intrinsic metabolic status of BC was responsible for GPR81 mediated glucometabolic reprogramming.

### Immunosuppression induced by GPR81-mediated enhanced glucose metabolism in BC from a transwell co-culture system

**Fig. 5:**
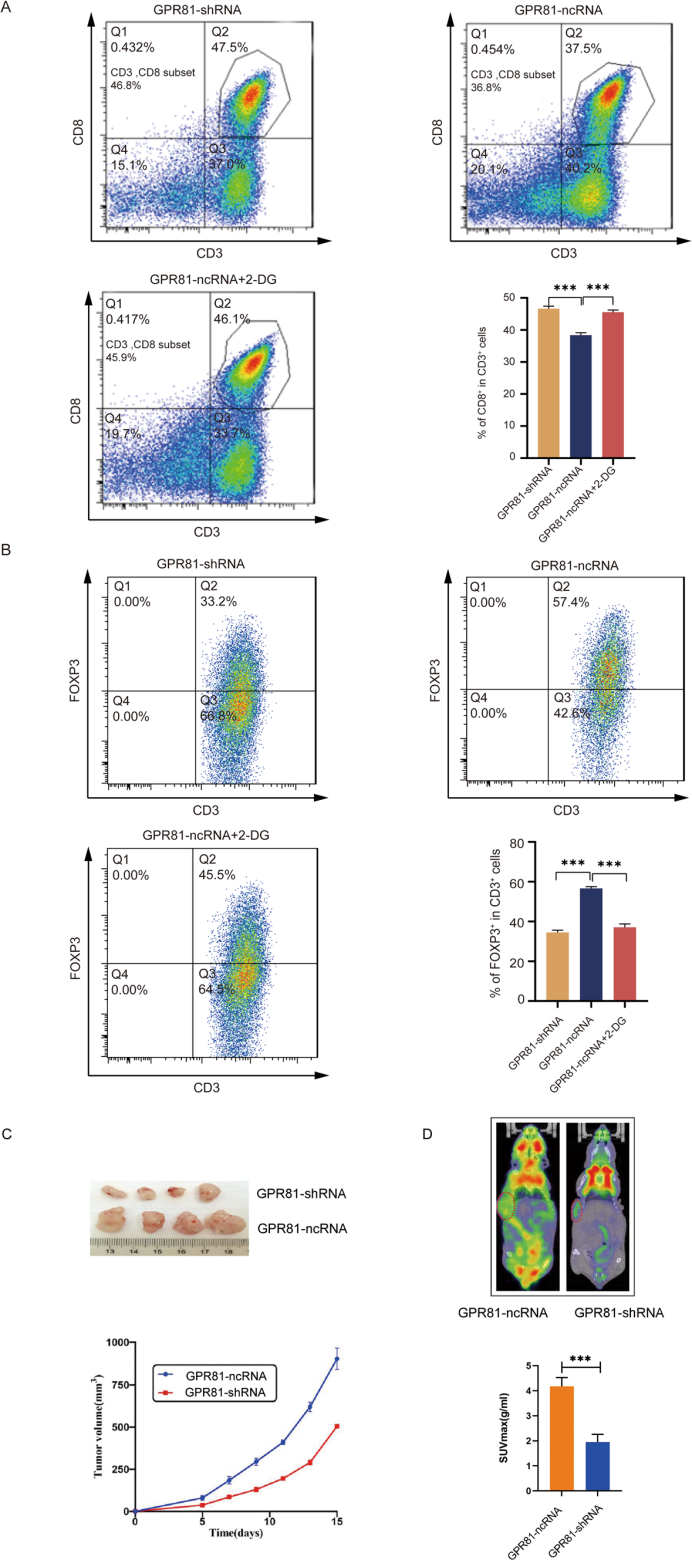
GPR81 mediated enhanced glycolysis led to an impaired immune response in BC. A transwell co-culture system was used to identify the crosstalk between GPR81-mediated glucometabolic reprogramming and immunoregulation. **A** Under polarizing conditions to form induced regulatory T cells, a remarkably decreased representation of CD3^+^CD8^+^ T cells was found in the co-culture system involving PBMCs and MDA-MB-231-GPR81-ncRNA in contrast with that in the co-culture system involving PBMCs and MDA-MB-231-GPR81-shRNA. Moreover, the addition of 2-DG to block the enhanced glycolysis mediated by GPR81 in the transwell co-culture system with MDA-MB-231-GPR81-ncRNA resulted in an increased percentage of CD3^+^CD8^+^ T cells. Both representative profiles of scatter plots and an summarization of the percentages of CD3^+^CD8^+^ T cells were shown. **B** Whereas, a markedly increased representation of CD3^+^FOXP3^+^ cells was observed in the co-culture system involving PBMCs and MDA-MB-231-GPR81-ncRNA in contrast with that in the co-culture system involving PBMCs and MDA-MB-231-GPR81-shRNA. In addition, 2-DG treatment in the transwell co-culture system with MDA-MB-231-GPR81-ncRNA resulted in a decreased percentage of CD3^+^FOXP3^+^ cells, both representative profile of scatter plots and summarization of the percentages of CD3^+^FOXP3^+^ T cells were shown. **C** The tumor volume-time growth curves showed that the xenografts derived from MDA-MB-231-GPR81-shRNA were dramatically inhibited compared to that derived from MDA-MB-231-GPR81-ncRNA. **D** ^18^F-FDG mirco-PET/CT imaging was performed to ascertain the relationship between ^18^F-FDG accumulation in vivo and GPR81 level in xenografts. ^18^F-FDG accumulation was decreased in xenograft with a lower SUVmax derived from MDA-MB-231-GPR81-shRNA compared to that with a higher SUVmax derived from MDA-MB-231-GPR81-ncRNA, suggesting inhibited glucose metabolism for xenografts derived from MDA-MB-231-GPR81-shRNA in vivo. *p < 0.05, **p < 0.01, ***p < 0.001

A transwell co-culture system involving BC cells and PBMCs was used to identify the crosstalk between GPR81-mediated glucometabolic reprogramming and immunoregulation. Under polarizing conditions to form induced regulatory T cells, the percentages of CD3^+^CD8^+^ T cells in PBMCs (Fig. 5A) significantly decreased, whereas the percentages of CD3^+^FOXP3^+^ cells in PBMCs (Fig. 5B) markedly increased after co-culturing of PBMCs with MDA-MB-231-GPR81-ncRNA cells in contrast with that after co-culturing with MDA-MB-231-GPR81-shRNA cells, suggesting an induced immunosuppression potentially medicated by GPR81 on BC cells. Moreover, the addition of 2-DG to BC cells before co-culturing to block the enhanced glycolysis mediated by GPR81 also resulted in improved immune landscape reflected by increased percentages of CD3^+^CD8^+^ T cells (Fig. 5A) and decreased percentages of CD3^+^FOXP3^+^ T cells (Fig. 5B) in transwell co-culture system involving PBMCs and MDA-MB-231-GPR81-ncRNA, which indicated crosstalk between immunomodulation and GPR81-mediated reprogramming of glucose metabolism in BC. Human BC xenograft models were also established to ascertain the role of GPR81 in glucometabolic reprogramming of BC in vivo. As indicated in the tumor volume-time growth curves (Fig. 5C), the xenografts derived from MDA-MB-231-GPR81-shRNA exhibited dramatically inhibited growths compared to that derived from MDA-MB-231-GPR81-ncRNA. Accordingly, ^18^F-FDG micro-PET/CT imaging demonstrated that the accumulation of ^18^F-FDG in xenografts was significantly correlated with GPR81 level, with a significantly lower SUVmax for MDA-MB-231-GPR81-shRNA than that for MDA-MB-231-GPR81-ncRNA (Fig. 5D).

## Discussion

Metabolic reprogramming and immune escape are increasingly accepted as significant hallmarks of tumors, and the intricate interplay between each other was already described previously [22, 23, 25]. Metabolomics analysis demonstrated that BC based on molecular subtype classification was characterized by distinct metabolic landscapes [27–30]. Furthermore, heterogeneity existing in BC with the same molecular subtype is partially attributed to different metabolic statuses [31]. As shown in our results, different local immune landscapes were classified in BC with different molecular subtypes. In contrast with BC with a Luminal subtype, BC with a non-Luminal subtype, particularly for BC with a TN subtype, was considered to have a high immunogenicity, resulting in more abundant TILs in TME. Given the relationship between tumor immune profile and tumor metabolic reprogramming, a biomarker associated with metabolic regulation is expected to predict the status of local immune microenvironment in BC and subsequently to select potentially responsive BC patients for ICB therapy.

GPR81, as a receptor for lactate, was previously reported as a prominent molecule associated with metabolism, angiogenesis and immunosuppression in BC [21, 24, 25, 32]. Nevertheless, the relationship between GPR81-mediated metabolic regulation and induced immunomodulation in BC was rarely described. The novelty of our investigation is to determine the interplay between the immune landscape in the tumor microenvironment (TME) and GPR81-mediated glucometabolic reprogramming of BC. As revealed in our study, glucometabolic reprogramming of BC through a GPR81-mediated Hippo-YAP signaling pathway was responsible for the distinct immune landscape in BC. In the present investigation, the relationship between GPR81 expression and local immune cell infiltration was first determined by IHC assay in breast cancer microarray tissues. Then, a series of cytological experiments in vitro were performed to identify the role of GPR81 in glucometabolic modulation and induced immune attenuation in BC. Whereas, previous reports from Ishihara et al. [21]. and Yang et al. [24] mainly aimed to assess the value of GPR81 in metabolic regulation in BC. Therefore, most of tests in their investigations were designed based on cytological experiments in vitro, human breast cancer tissue-derived results were rare. Moreover, only triple-negative (TN) BC cell line was used in the two reports mentioned above, but a total of three human BC cancer cell lines were used in our study, including T47D, SK-BR-3, MDA-MB-231, which represent BC with a Luminal subtype, a HER2 ( +) subtype, and a TN subtype, respectively. As shown in our results, the intrinsic metabolic status of each molecular subtype of BC cells was distinct from each other, which potentially led to the different participation of the Hippo-YAP signaling pathway in glucometabolic reprogramming mediated by GPR81 in BC. In other words, the relatively detailed and more complex molecular mechanism responsible for glycolytic regulation in BC identified in our study was also a significant novelty in contrast with other previous researches. Although aa study from Brown et al. [25] focused on the role of GPR81 in the interaction between immune regulation of BC and BC growth, they only emphasized on a paracrine mechanism responsible for the binding of lactate with GPR81 expressed on the antigen-presenting cell (APC) but not on BC cells, and GPR81 mediated metabolism regulation was absent in their results.

**Fig. 6:**
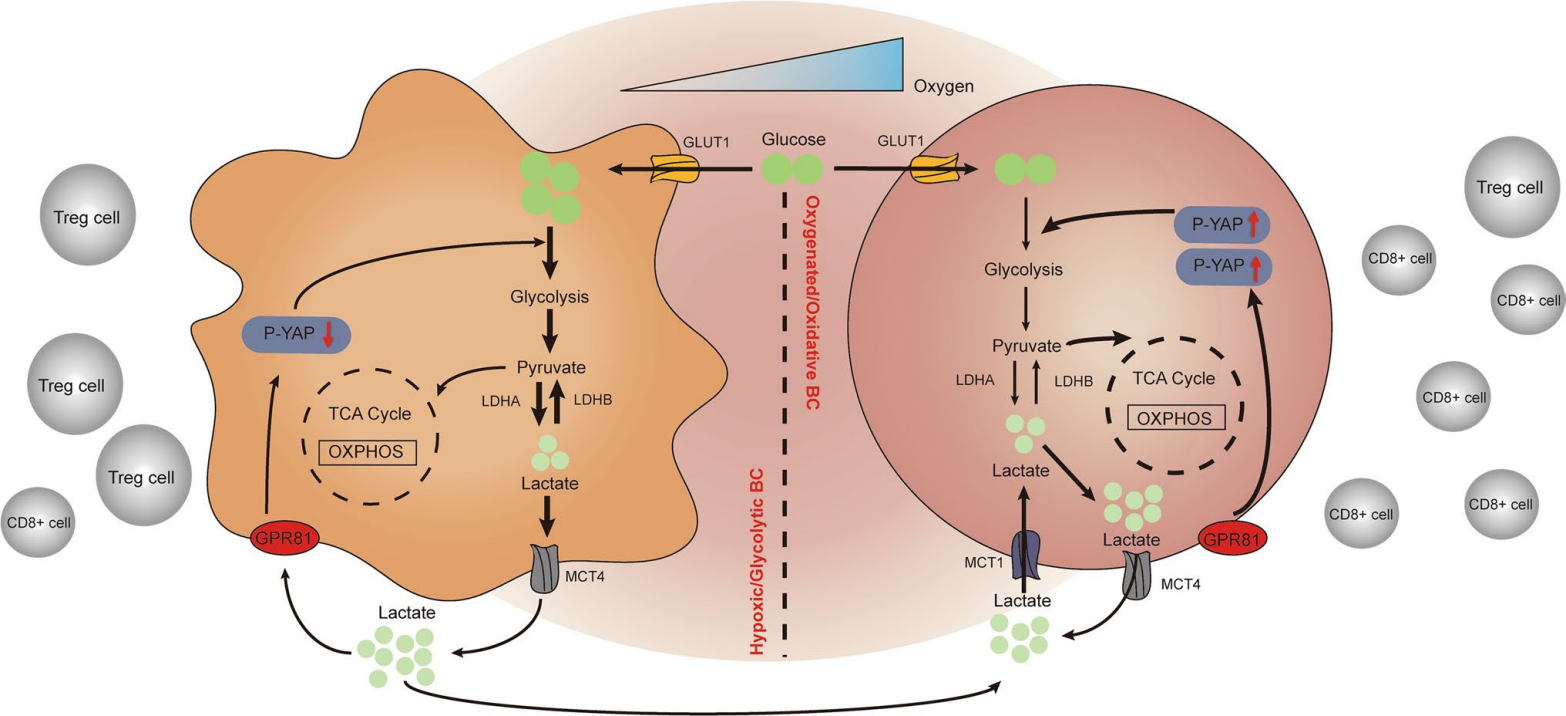
A hypothetical scheme of the interplay between the surrounding immune landscape and GPR81-mediated YAP signaling dependent BC glucometablic reprograming. For hypoxic/glycolytic BC under hypoxia, increased lactate production induced by enhanced glycolysis tends to further promote glycolysis by interaction with GPR81 in a positive feedback manner, which is dependent on a decreased level of YAP phosphorylation. Consequently, the surrounding CD8^+^ T cell infiltration is dramatically decreased, whereas the Treg infiltration is remarkably increased. For oxygenated/oxidative BC cells under sufficient oxygen, lactate accumulation in the local microenvironment is inclined to be transported by monocarboxylate transporter 1 (MCT1) into the inside of BC cells to go through oxidative phosphorylation via tricarboxylic acid (TCA) cycle, which is dependent on an increased level of YAP phosphorylation. In contrast to the status in hypoxic/glycolytic BC, the CD8^+^ T cell infiltration in the TME with sufficient oxygen is significantly enhanced, whereas the Treg infiltration is markedly decreased

IHC analysis in our present investigation indicated that TIL status in BC was associated with GPR81 expression. As described in the results, BC with a TN subtype expressing a higher level of GPR81 tended to exhibit attenuated anti-tumor immune response (lower ratios of CD8/FOXP3) in comparison with that expressing a lower level of GPR81. Meanwhile, glycolytic metabolism detection in BC cells in vitro confirmed the role of GPR81 in the regulation of glucose metabolism. Mechanistically, a Hippo-YAP signaling dependent on the intrinsic status of glucose metabolism in BC cells was considered one of the molecular mechanisms for GPR81-mediated glycolytic reprogramming [33]. As reported previously, glucose metabolism and YAP activity were closely connected [34, 35]. Inhibition of glucose uptake and glycolysis, or a shift from aerobic glycolysis to oxidative phosphorylation, resulted in a remarkable blockade of YAP activity, suggesting glycolysis is required for full transcriptional activity of YAP by promoting interaction between YAP and TEAD, which is mediated by some key glycolysis enzyme, such as PFK1 [33]. In turn, YAP also mediated the regulation of glucose metabolism by directly or indirectly enhancing expression of the key glycolysis proteins or enzymes, such as GLUT-3 and HK2 [34]. Collectively, these findings indicated that there might be a positive feedback loop between glycolysis and YAP activity. In other words, YAP activity was dependent on the intrinsic status of glucose metabolism in tumor cells. As shown in the results, for the relatively glycolytic breast cancer cell line with a TN subtype (MDA-MB-231) which was characterized by enhanced glycolysis, lactate-GPR81 interaction mediated enhanced YAP activity (deceased phosphorylation of YAP (p-YAP)) by inhibition of Hippo-YAP signaling). Conversely, the downregulation of GPR81 in glycolytic MDA-MB-231 cells led to decreased glycolysis dependent on the activation of Hippo-YAP signaling with an increased p-YAP. In contrast, in a relatively oxidative breast cancer cell line with a Luminal subtype (T47D), the glycolytic activity was not remarkably reduced by the downregulation of GPR81. Additionally, lactate-GPR81 interaction mediated inhibition of YAP activity (increased p-YAP by activation of Hippo-YAP signaling). And the downregulation of GPR81 resulted in enhanced YAP activity with deceased p-YAP by inhibition of Hippo-YAP signaling. Presumably, lactate addition to oxidative T47D cell prompted lactate transport into cells instead of glucose uptake by cells to elicit oxidative phosphorylation. A hypothetical model of the interplay between the surrounding immune landscape and GPR81-mediated YAP signaling-dependent glucometablic reprograming based on the intrinsic metabolic status of BC were schemed in Fig. 6.

In contrast with BC with a Luminal subtype, BC with a TN or a HER2 subtype is more immunogenic and is capable of initiating an local anti-tumor immune response. Consistently, a higher TIL score is always expected for BC with a TN or a HER2 subtype [36, 37]. From a perspective of homeostasis, immune balance in vivo is maintained by both positive immune response and negative immunosuppression. However, this balance mechanism is hijacked by tumors to achieve immune escape [38, 39]. Presumably, the “immune hot” nature characterized by an simultaneous increase in PD-L1 expression and TIL score in BC with a TN or a HER2 subtype result from the enhanced local anti-tumor immune response (IFN-γ secretion by surrounding immune cells) triggered by the strong immunogenicity of BC and subsequent induced expression of PD-L1 on tumor cells mediated by IFN-γ signaling to inhibit potentially excessive immune response [40, 41]. Accordingly, tumor immunotherapy, such as ICB therapy, holds promise for neoadjuvant therapy for BC with a TN or a HER2 subtype [42, 43]. However, the efficiency of ICB for BC in clinic is far from satisfying, which is mainly caused by the heterogeneous immune landscapes. The established transwell co-culture system in our study allowed us to directly explore the interplay between the local immune landscape and GPR81-mediated glycolytic reprogramming in BC. Promisingly, the determined relationship between the local immunological TME remodeling and GPR81-mediated metabolic regulation in BC in our investigation is able to provide a feasible approach to stratify BC patients to maximize the efficacy of ICB therapy. GPR81 is suggested as an effective biomarker to screen potentially sensitive BC patients to ICB therapy, especially for BC with a TN subtype [44]. Mechanistically, as previously reported, a competition for glycolytic metabolism between BC cells and surrounding lymphocytes at least partially led to impaired local immune response against BC [45].

Despite the aforementioned exciting results, several limitations with respect to the present investigation is of quite necessity to be addressed. First, TIL representation in BC tissue samples was only evaluated by IHC staining, an immunologic function assay was not performed to analyze the functional status of immune cells in the local TME. Isolation of TILs from fresh BC tissue samples and subsequent cytometric analyses would partially help to ascertain this issue. Second, apart from the established transwell co-culture system in vitro, no in vivo animal models and experiments were available to confirm the direct correlation between GPR81-mediated reprogramming of glucose metabolism and induced immunosuppression in TME. Fortunately, IHC staining results from BC microarray chips tissues at least partially addressed this issue in vivo. In the end, the predictive role of GPR81 as a biomarker in ICB therapy for BC was not directly detected in or preclinical models or clinical settings, which is warranted in our future study.

## Conclusion

Taken together, glucometabolic reprogramming of BC through a GPR81-mediated Hippo-YAP signaling pathway contributed to the distinct immune landscape in BC. On the one hand, our investigation provided an innovative perspective to understand the complex microenvironment in BC, especially for the interplay between BC cells and the surrounding immune cells. On the other hand, GPR81 was suggested as a potential biomarker to screen responsive BC patients prior to ICB therapy and as a potential target to make a comprehensive treatment strategy involving immunotherapy and metabolism intervention.

## Supplementary Information

Additional file1 (RAR 230 KB)

## Data Availability

The datasets used and/or analyzed during the current study are available from the corresponding author upon appropriate request.
